# Electro-optic spatial decoding on the spherical-wavefront Coulomb fields of plasma electron sources

**DOI:** 10.1038/s41598-018-21242-y

**Published:** 2018-02-13

**Authors:** K. Huang, T. Esirkepov, J. K. Koga, H. Kotaki, M. Mori, Y. Hayashi, N. Nakanii, S. V. Bulanov, M. Kando

**Affiliations:** 1 Kansai Photon Science Institute, National Institutes for Quantum and Radiological Science and Technology (QST), 8-1-7 Umemidai, Kizugawa-city, Kyoto 619-0215 Japan; 2 Institute of Physics ASCR, v.v.i. (FZU), ELI BEAMLINES, Za Radnicí 835, Dolní Břežany, 252241 Czech Republic

## Abstract

Detections of the pulse durations and arrival timings of relativistic electron beams are important issues in accelerator physics. Electro-optic diagnostics on the Coulomb fields of electron beams have the advantages of single shot and non-destructive characteristics. We present a study of introducing the electro-optic spatial decoding technique to laser wakefield acceleration. By placing an electro-optic crystal very close to a gas target, we discovered that the Coulomb field of the electron beam possessed a spherical wavefront and was inconsistent with the previously widely used model. The field structure was demonstrated by experimental measurement, analytic calculations and simulations. A temporal mapping relationship with generality was derived in a geometry where the signals had spherical wavefronts. This study could be helpful for the applications of electro-optic diagnostics in laser plasma acceleration experiments.

## Introduction

With the advantages of high acceleration gradient ($\sim GV/cm$) and ultra-short temporal structure ($\sim fs$), laser wakefield acceleration (LWFA)^1^ has been investigated intensively in the past decade^2–11^. Applications of the laser wakefield accelerated electron beams and LWFA based secondary x-ray sources^12–16^ for pump-probe studies have been proposed^17,18^. Concerning applications, temporal information of the electron beam could be important because it has an impact on the resolution of the experiment. Previous studies on electron temporal diagnostics were performed by detecting and analyzing the coherent transition radiation (CTR) produced by electron beams^19–21^. Although electron pulse durations of 50 fs to 1.7 fs (rms) have been demonstrated, such measurements either lacked single-shot capability because of low conversion efficiency from electron to terahertz (THz) radiation, or sacrificed electron beam quality by inserting a metal radiator in the beam path. On the other hand, none of these experiments have shown electron arrival timing results which are essential for determining the temporal fluctuation of the electron beams.

Electro-optic (EO) sampling is a widely used technique in conventional accelerators^22–34^. When placing an EO crystal close to the electron beam path, the Coulomb fields of electrons residing in the THz range propagate through the crystal and act as a DC bias inducing the Pockels effect. By the incidence of a probe laser through the EO crystal at similar timing, the electron Coulomb field is converted to birefringence. In this way, the electron temporal information can be detected non-destructively in a single shot. The maximum phase retardation is achieved as ${{\rm{\Gamma }}}_{max}={n}_{0}^{3}{r}_{41}{E}_{THz}{\omega }_{0}d/c$ when setting the [−1,1,0] axis of the crystal parallel to the Coulomb field polarization direction, where *n*_0_ is the refractive index of probe laser in EO crystal, *r*_41_ is the EO coefficient, *ω*_0_ is the probe laser frequency, *d* is the crystal thickness and *c* is the speed of light. Methods such as electro-optic spectral decoding (EOSD)^23^ and electro-optic temporal decoding (EOTD)^24^ have been invented to measure the electron pulse duration down to <100 fs^25,26^.

For the measurement of the electron arrival timing jitter, a spatial decoding method has been introduced from THz studies^27^. It has a simple set-up where the probe beam is incident on the crystal with an angle relative to the electron beam path^28–33^ so that the temporal profile of the electron Coulomb field is transversely recorded on the probe pulse. Usually a cross-polarizer set-up is used to pick out the polarization rotated signal. A measured signal down to 82 fs (rms) has been demonstrated in a conventional accelerator^30^. Also, this diagnostic served well as a simple electron beam timing monitor detecting the arrival time of the signal peak^28–32^. Previously, a model has been widely used by considering the Coulomb field compressed in a narrow cone perpendicular to the electron moving direction. The wavefront of the Coulomb field signal has an incident angle of *θ*_*s *_= 0 to the EO crystal surface. In such a geometry, a linear temporal mapping relationship *c*Δ*τ* = Δ*ξ* tan *θ*_*p*_ was utilized to deduce the timing information from the measured EO signal^28,30,33,35^, where Δ*τ* is the timing difference, Δ*ξ* denotes the observed displacement on the CCD and *θ*_*p*_ is the relative angle between probe laser and electron beams. Up to now, non-destructive single-shot EO spatial decoding diagnostics and the corresponding temporal mapping relationship in LWFA have not yet been investigated.

In this paper, we introduced the EO spatial decoding diagnostics to LWFA. By placing the EO crystal very close to the electron source, the measured temporal mapping relationship was found to be inconsistent with that used in previous work. Analytical calculations and simulations revealed that the inconsistency was due to the spherical structure of the Coulomb field when a relativistic electron beam was very close to the source. A general nonlinear temporal mapping relationship was derived in a geometry where the signals had spherical wavefronts. We believe this work could be helpful for applications of the EO spatial decoding method in laser plasma acceleration experiments.

## Results

### EO diagnostics set-up

**Figure 1:**
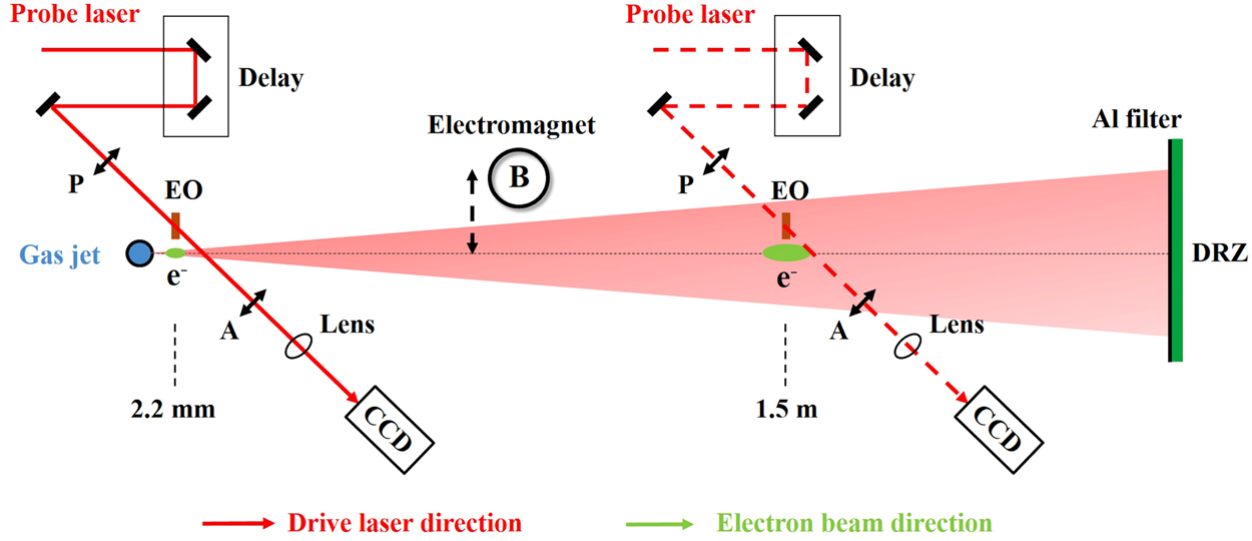
Schematic of EO detection. The electron beam temporal profiles were measured at two different positions with longitudinal distances of 2.2 mm and 1.5 m from the gas jet exit. P and A denote a pair of polarizers with polarization direction orthogonal to each other. A removable electromagnet was used to measure the electron energy. Both the EO signals and electron spectra signals were recorded by 16 bit CCDs coupled with optical imaging systems. The timing of the probe laser can be continuously changed via a delay stage. The electron beams passed through a removable electromagnet coupled with a 2 mm lead slit and were recorded on a Gd_2_O_2_S: Tb phosphor screen (Mitsubishi Chemical, DRZ-High). A 100 *μ*m Al filter was placed in front of the DRZ to block visible light.

A 10 TW, 40 fs (FWHM) drive laser was focused by an F/20 off-axis-parabola mirror to a 3 mm conical Helium gas jet to generate relativistic electrons (see Methods for details). To perform EO sampling, a 50 *μ*m thick, (110) cut GaP crystal was utilized with the [−1, 1, 0] optical axis perpendicular to the propagation direction drive laser. The 40 fs (FWHM) probe laser was incident through the EO crystal to perform spatial decoding. The relative angle between probe laser and drive laser was set to be *θ*_*p*_ = 44°. We noted that, different from conventional accelerators, there is always a residual intense drive laser together with the electron bunch on the beam path as shown in Fig. 1.

The EO crystal could be damaged by the strong irradiance of the transmitted drive laser pulse. The damage threshold of the GaP crystal by a femtosecond laser has been measured to be ∼ 4.3 mJ/cm^2^ ^36^. Since the electric field of the electron beam has an opening angle of 2/*γ*^33^, the fields get broader and weaker with larger transverse distance. The EO crystal should be placed very close to the drive laser axis. In the experiment, the detection point of the probe laser (probe center) on the EO crystal was set at a distance of 1.5 mm from the laser axis. Considering a 400 mJ laser pulse and F/20 light cone, the GaP crystal should be placed at least 2 m away from the target to avoid damage. However, even when we put the EO crystal 1.5 m away from the target, the EO signals were barely observed in the experiment. The reasons include: (1) Laser wakefield accelerated electron bunches always have energy spreads magnitudes broader than that from conventional accelerators. For large propagation distance, the electron bunch durations will be elongated because of the propagation time lag between electrons with different energies. The peak currents will drop drastically; (2) Existing divergences of several mrad level result in an electron spot size of ∼ cm at large propagation distance. The charge densities could drop by several orders of magnitude; (3) Due to pointing fluctuation, the electron beam could arrive at positions with transverse distance so large to the EO crystal that the field strength is too weak to be detected.

**Figure 2:**
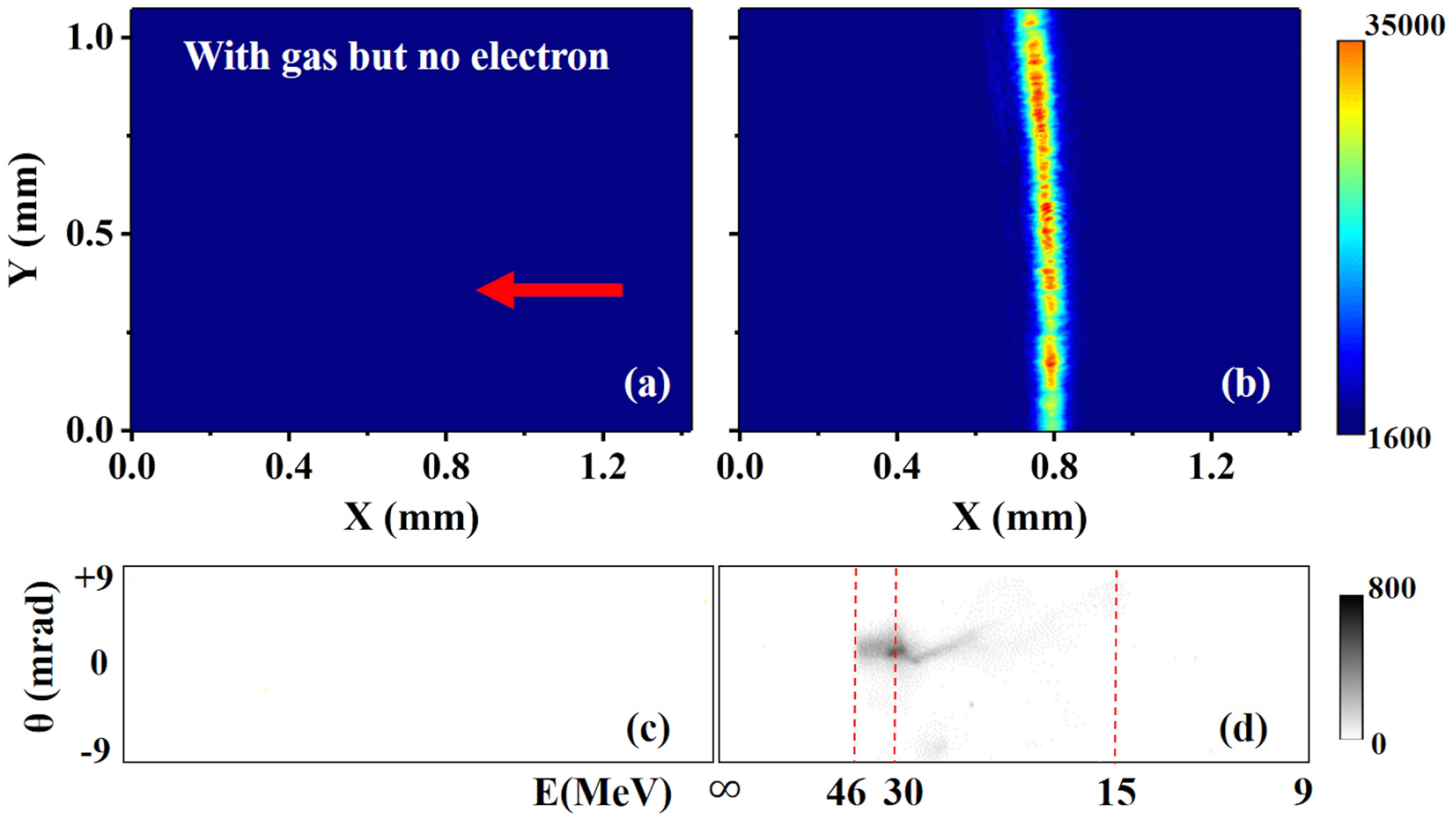
Typical EO signals and corresponding electron energy spectra. EO signals are shown in: (**a**) when no electron beams were generated at lower plasma density; (**b**) when electron beams were generated at a higher plasma density. The corresponding electron spectra are illustrated in (**c**) and (**d**), respectively. The X and Y coordinates in (**a**) and (**b**) denote the measured displacement on the CCD. The red arrow illustrates the signal arrival timing direction. The color bars denote the signal intensities on the CCDs. In the experiment, the background EO signal intensity is ∼1600.

A solution was found by placing the EO crystal very close to the target. As shown in Fig. 1, the crystal was at a position 2.2 mm downstream of the target exit. With similar transverse displacement, the crystal could reside at a position outside of the light cone of the transmitted drive laser. Also, at such a distance, the electric field weakening resulting from the electron beam divergence and energy spread is negligible. Figure 2 shows the typical experimental results of EO signals and corresponding electron energy spectra signals. To check the source of the EO signal, we took data in three different cases: (i) without gas; (ii) with gas but no electron beams were observed; (iii) electron beams were generated. Data in case (i) was used as background. As for case (ii), we found that when the gas densities were relatively low, no electron beams were observed, which suggested that the wakefield strengths were below the self-injection threshold^3^. Simultaneously, no EO signals were observed, as illustrated in Fig. 2(a) and (c). For case (iii), when electron signals appeared at a slightly higher density (${n}_{e} > 2.0\,\times \,{10}^{19}$ cm^−3^), EO signals were also observed, as shown in Fig. 2(b). The corresponding electron energy spectrum is shown in Fig. 2(d). This comparison demonstrated that the EO signals were generated by the electric field accompanying the electron beams.

### Measurement of temporal mapping relationship

The typical EO signal profiles showed a curved structure, as illustrated in Fig. 2(b), which means that the upper and lower parts of the signal in the vertical direction have a slight arrival time difference on the EO crystal. This suggested that the wavefront of the Coulomb field is not completely a plane wave. To verify the Coulomb field propagation direction, we checked the signal displacement dependence on various probe laser delay settings at a plasma density of 3.5 × 10^19^ cm^−3^ where electron beams with relatively stable timings were generated. The electron beams had pointing fluctuations residing in ±10 mrad and divergences of (${4.6}_{-1.1}^{+1.6}$, ${5.1}_{-1.0}^{+1.8}$) mrad (FWHM) in the horizontal and vertical directions. Since the EO crystal was placed 2.2 mm away from the plasma exit, the detection of the temporal mapping relationship was barely affected by such small fluctuations.

**Figure 3:**
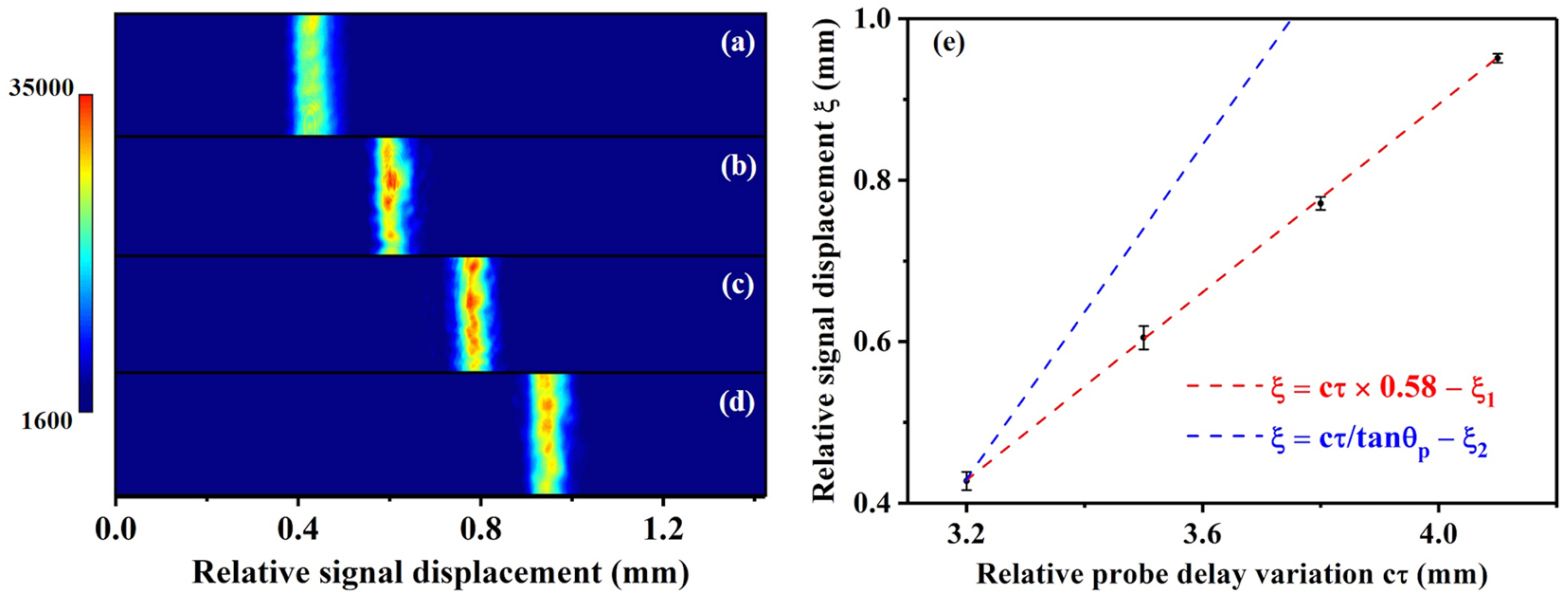
Temporal mapping relationship measurement. The EO signal profiles of the same vertical region at four different probe delays are illustrated in (**a**–**d**). The signals shifted from left to right with similar intervals. Zero position of the relative signal displacement denotes the left edge of the CCD chip. Statistics of the EO signal and linear fitting of the temporal mapping relationship are shown in (**e**). The red dashed line denotes a linear fitting of the experiment results, while the blue dashed line illustrates a plot based on a previously used temporal mapping relationship *c*Δ*τ* = Δ*ξ* tan *θ*_*p*_.

The EO signals were taken at four different probe delays: 3.2 mm, 3.5 mm, 3.8 mm, 4.1 mm (relative value), as shown in Fig. 3(a–d), respectively. To eliminate shot by shot fluctuations, statistics on the peak positions of 20 consecutive EO signals at each probe delay were made, as shown in Fig. 3(e). The mean positions are denoted by black dots and standard deviations are denoted by error bars. From this measurement, we can deduce the temporal mapping relationship between the signal displacement *ξ* and timing variation *cτ* by linear fitting, as illustrated by the red dashed line in Fig. 3(e). The fitted relationship was *ξ* = 0.58*cτ* − *ξ*_1_, where *ξ*_1_ represents a relative initial value. Thus, the measured temporal mapping relationship can be written as *c*Δ*τ* = 1.72Δ*ξ*. To our surprise, the slope 0.58 of the fitted red dashed line was much smaller than 1/tan *θ*_*p*_ ≈ 1.04 based on *c*Δ*τ* = Δ*ξ* tan *θ*_*p*_, as illustrated by the blue dashed line set with the same beginning point in Fig. 3(e). This measurement is somehow inconsistent with the previous assumption that the Coulomb field of the relativistic electron beam is compressed as a narrow cone perpendicular to the electron path and propagates along the electron moving direction^22–26,28–33^. The smaller slope of the plot implied that the relative angle between the probe laser and Coulomb field wavefront propagation direction is much larger than *θ*_*p*_.

### Calculation of the Liénard–Wiechert potential

**Figure 4:**
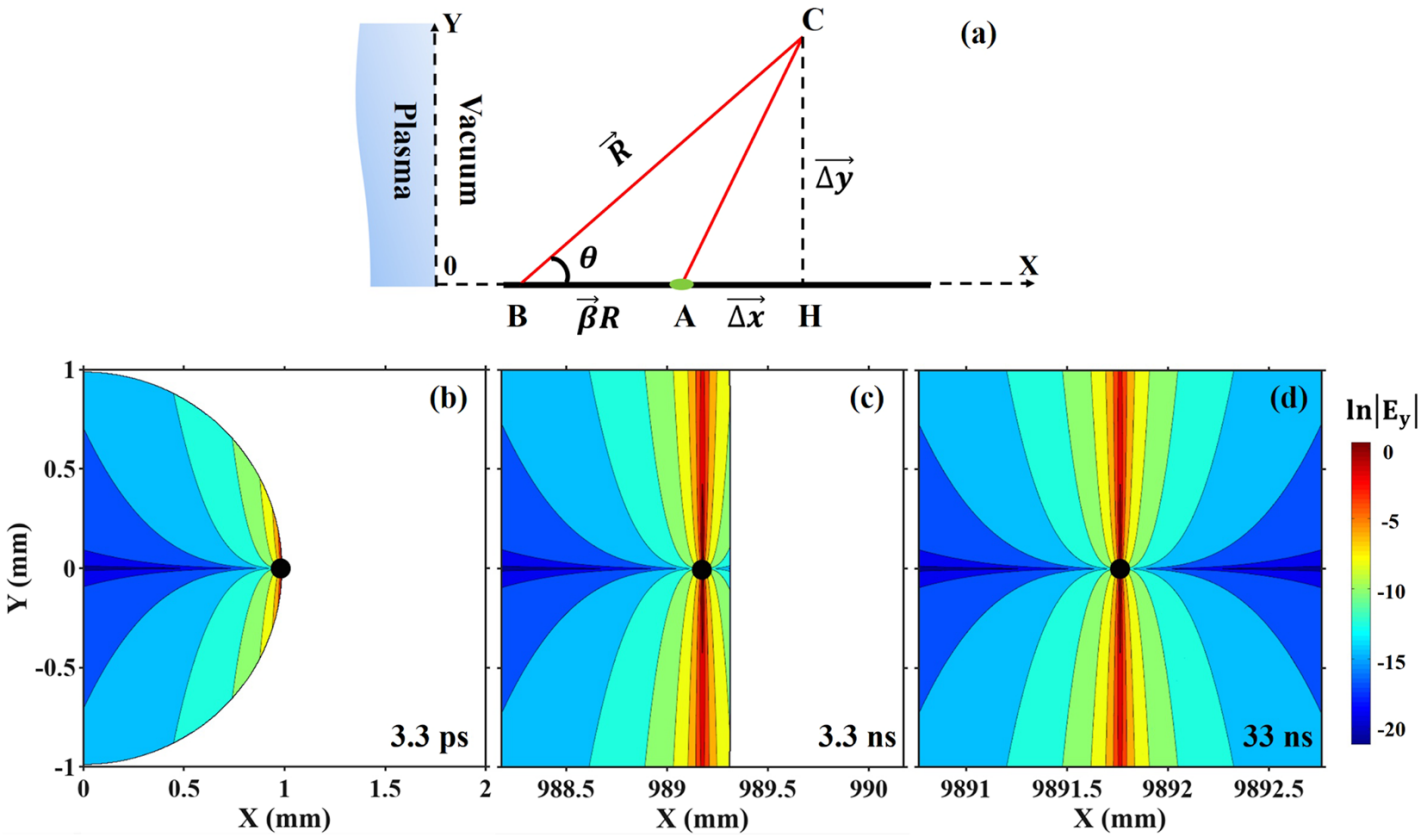
Calculation of the Coulomb field of a single electron. (**a**) illustrates the calculation scheme. The vector $\overrightarrow{AH}$ and $\overrightarrow{HC}$ can be represented as $\overrightarrow{{\rm{\Delta }}{\rm{x}}}$ and $\overrightarrow{{\rm{\Delta }}{\rm{y}}}$; (**b–d**) show the **ln**|*E*_*y*_| distributions in an area of 2 mm × 2 mm at electron propagation times of 3.3 ps, 3.3 ns and 33 ns, respectively. The black dots in (**b–d**) denote the electron postions at corresponding timings.

To understand the physics of how the Coulomb field evolves, we calculated the Liénard–Wiechert potential of a single electron with an energy of 30 MeV. The calculation scheme is shown in Fig. 4(a). The electron emitted from “0” position propagates along the *x* direction with a velocity of *βc*, while the plane *x* = 0 denotes the boundary between the plasma and vacuum. The electric field can be calculated as^37^

1 $$\overrightarrow{E}=-\frac{e}{4\pi {\varepsilon }_{0}}{[\frac{\overrightarrow{n}-\overrightarrow{\beta }}{{\gamma }^{2}{(1-\overrightarrow{\beta }\cdot \overrightarrow{n})}^{3}{R}^{2}}]}_{ret}=-\frac{e}{4\pi {\varepsilon }_{0}}{[\frac{\overrightarrow{AH}+\overrightarrow{HC}}{{\gamma }^{2}{(1-\overrightarrow{\beta }\cdot \overrightarrow{n})}^{3}{R}^{3}}]}_{ret}$$

where *ε*_0_ is the vacuum permittivity, “*ret*” means the field is calculated at a retarded time, *R* is the retarded source to observer distance, $\overrightarrow{n}=\overrightarrow{R}/R$ and *γ* = 1/(1 − *β*^2^)^1/2^. Assuming that at time t, the electron is at position A (*βct*, 0). The electric field at point C(X, Y) was generated at an earlier time when the electron was at position B. We have $|\overrightarrow{BC}|\,=R$ and $|\overrightarrow{BA}|\,=\beta R$. The retarded source to observer distance *R* can be calculated by the equation (*βR* + Δ*x*)^2^ + Δ*y*^2^ = *R*^2^(*R* > 0), where Δ*x* = *X* − *βct* and Δ*y* = *Y*. Thus, we get: $R(X,\,Y,\,t)=\beta {\rm{\Delta }}x{\gamma }^{2}+\sqrt{(\beta {\rm{\Delta }}x{\gamma }^{2}{)}^{2}+{\gamma }^{2}({\rm{\Delta }}{x}^{2}+{\rm{\Delta }}{y}^{2})}$.

The electric fields are shielded when the electron is inside the plasma. That is, when the source position B(*βct* − *βR*,0) is on the left side of the Y axis, the field strength at observer point C should be 0. Thus, the transverse electric field distribution of the whole space at a certain time *t* can be written as:

2 $${E}_{y}(X,Y,t)=\{\begin{array}{cc}-\frac{e}{4\pi {\varepsilon }_{0}}\,{[\frac{Y}{{\gamma }^{2}{R}^{3}{(1-\beta \cos \theta \cdot sgn(X-\beta ct+\beta R))}^{3}}]}_{ret}, & \beta ct-\beta R\ge 0.\\ 0, & \beta ct-\beta R < 0.\end{array}$$

where *sgn* is the sign function with values of *sgn*(*x*) = (1, x > 0; 0, x = 0; −1, x < 0) and *θ* is defined by sin $\theta =\,|\overrightarrow{{\rm{\Delta }}{\rm{y}}}|/R$. The calculated results of Eq. (2) are shown in Fig. 4(b–d).

When observing the overall electric field at a large angle and a small longitudinal distance, the difference of propagation distances between the field and the electron is so small that the front boundary of the Coulomb field is “attached” to the electron. The Coulomb field of the electron distributed along the spherical boundary with center at (0, 0), as shown in Fig. 4(b). This corresponds to the case in our experiment where the longitudinal distance between source and the crystal is 2.2 mm and the observation angle is 1.5 mm/2.2 mm ∼ 680 mrad. However, if the electric field is detected at a small angle 1 m away (1 mm/1 m = 1 mrad), as illustrated in Fig. 4(c), the curvature is negligible. Since the electron velocity is smaller than c, the field boundary is in front of the electron after propagating for certain distances. When the detection point is further away, as shown in Fig. 4(d), the boundary of the field is far from the electron. The Coulomb field is symmetrically confined as a cone perpendicular to the electron path. Figure 4(d) corresponds to previous work in conventional accelerators, where the EO crystals were placed several mm aside from the beam path while the electron beams had propagated hundreds of meters away from the cathode. In such cases, the electron beams can be considered as propagating in a free space. In our calculation, the electron suddenly appeared with the energy of 30 MeV, thus the rising edge looks like a cut-off. In a realistic case, the electron has an acceleration process inside the plasma and the rising edge of the field should be gentle. But still, the Coulomb field should be confined along the hemisphere with radius of *ct* at small propagation distances, where *t* is the electron propagation time.

### Simulations of the Coulomb field

To qualitatively study the propagation of the Coulomb field of electron beam and confirm the elaborated physics process in Fig. 4, we performed simulations using the code REMP^38^ (see Methods for details). We note that to understand the physics of how the spherical wavefront Coulomb field appears, it is unnecessary to repeat a specific electron parameter in the experiment. Thus, we focus on investigating the evolution of the electric field accompanying the electron bunches when they are propagating out of the plasma (first simulation) and passing by the EO crystal at 2.2 mm away from the source (second simulation).

**Figure 5:**
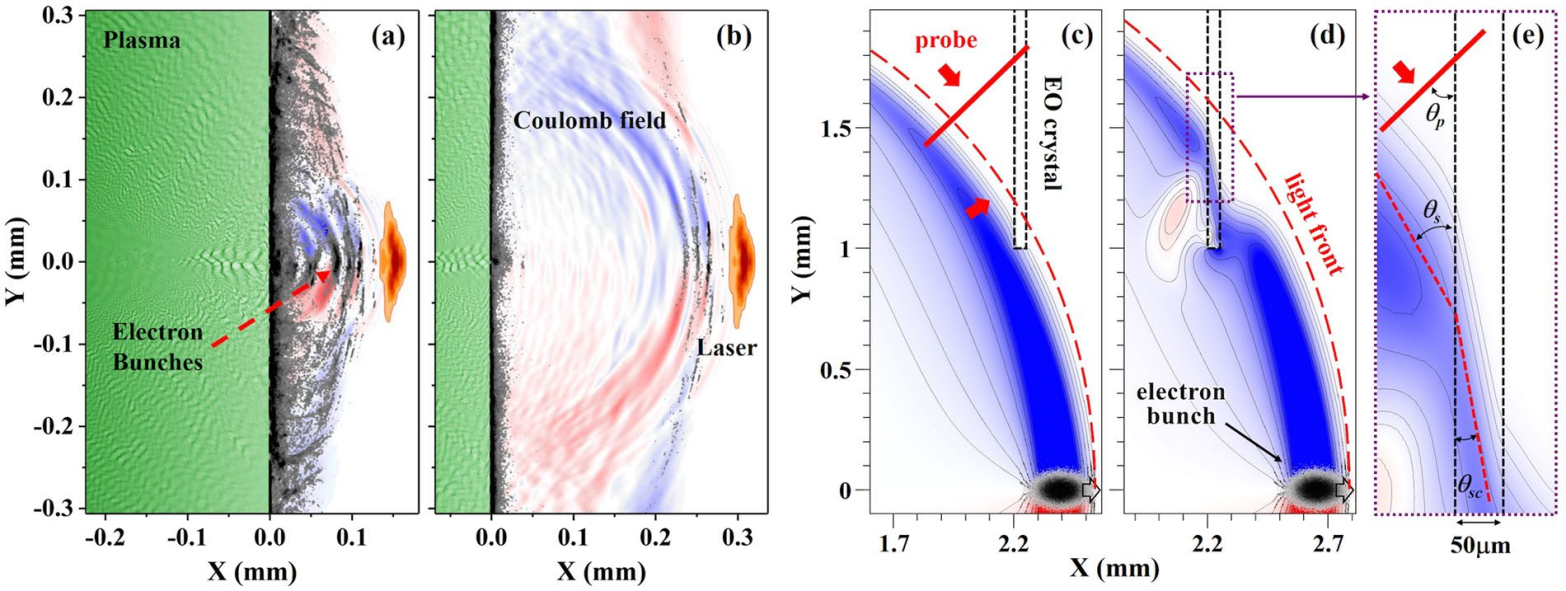
Two sets of simulation results for evolution of the Coulomb field. The first simulation (**a,b**) includes a full laser plasma interaction while the second one (**c–e**) models a simplified case where an electron bunch is propagating in vacuum. The “0” positions in the x direction of the figures are the plasma-vacuum boundary. (**a**) and (**b**) correspond to simulation times of 7.21 ps and 7.74 ps that are counted from the beginning of the laser interaction with plasma. The laser pulse is denoted by orange colour. The *E*_*y*_ component of low frequency electric fields are illustrated by blue and red colours corresponding to different field directions. The green area is the plasma bulk and electrons outside the plasma region are shown as black dots. (**c**) and (**d**) show the *E*_*y*_ field structures at times of 8.01 ps and 8.84 ps that are counted from the emergence of the electron bunch at the left boundary of the simulation box. The spherical red dashed curves in (**c**) and (**d**) denote an imagined light front expanding at the speed of *c*. A zoomed picture of the purple dashed rectangular area in (**d**) is shown in (**e**). In a small area, the angle of incidence and angle of refraction of the Coulomb field are denoted as *θ*_*s*_ and *θ*_*sc*_, respectively. The red dashed lines in (**e**) represent a signal wavefront in the field.

First, we considered the laser pulse interaction with a supersonic jet modeled as a plasma slab. The laser pulse propagated through the plasma producing several bunches of relativistic electrons. (see Methods for details.) The relativistic electrons escaping from the plasma were accompanied by their Coulomb field with a characteristic spherical distribution, as shown in Fig. 5(a). When electron bunches were in the plasma, the Coulomb fields were completely shielded. Therefore the Coulomb field distribution of emitted electrons evolves as it had been created at the plasma-vacuum interface due to the loss of quasi-neutrality. When the electron bunches propagated further away, the Coulomb fields expanded as spherical waves, as illustrated in Fig. 5(b). The observation of the spherical-wavefront Coulomb field in the simulation justifies our calculation of the Liénard–Wiechert potential above.

For comparison and further confirmation of the physics model, we performed a second simulation of an isolated electron bunch detaching from a neutralizing background (see Methods for details). In this simulation, we investigated on the Coulomb field shape when the electron beam passed by the EO crystal. Figure 5(c–e) show the electric field patterns when a 30 MeV electron beam passes by the EO crystal. We observed that the Coulomb field wavefront distributed as a spherical shape along an imagined light front with a radius of *ct* (*t* is the electron propagation time) centered at the electron emitting point. In a small area shown in Fig. 5(e), the signal could be considered as a plane wave with an incident angle of *θ*_*s*_ ≈ tan^−1^(1.5 mm/2.2 mm) = 34.3°. The relative angle between the probe laser and the signal wavefront propagation direction is larger than *θ*_*p*_. This is the reason why the measured slope of the temporal mapping relationship in Fig. 3(e) is much smaller than 1/tan *θ*_*p*_.

### Nonlinear temporal mapping relationship

**Figure 6:**
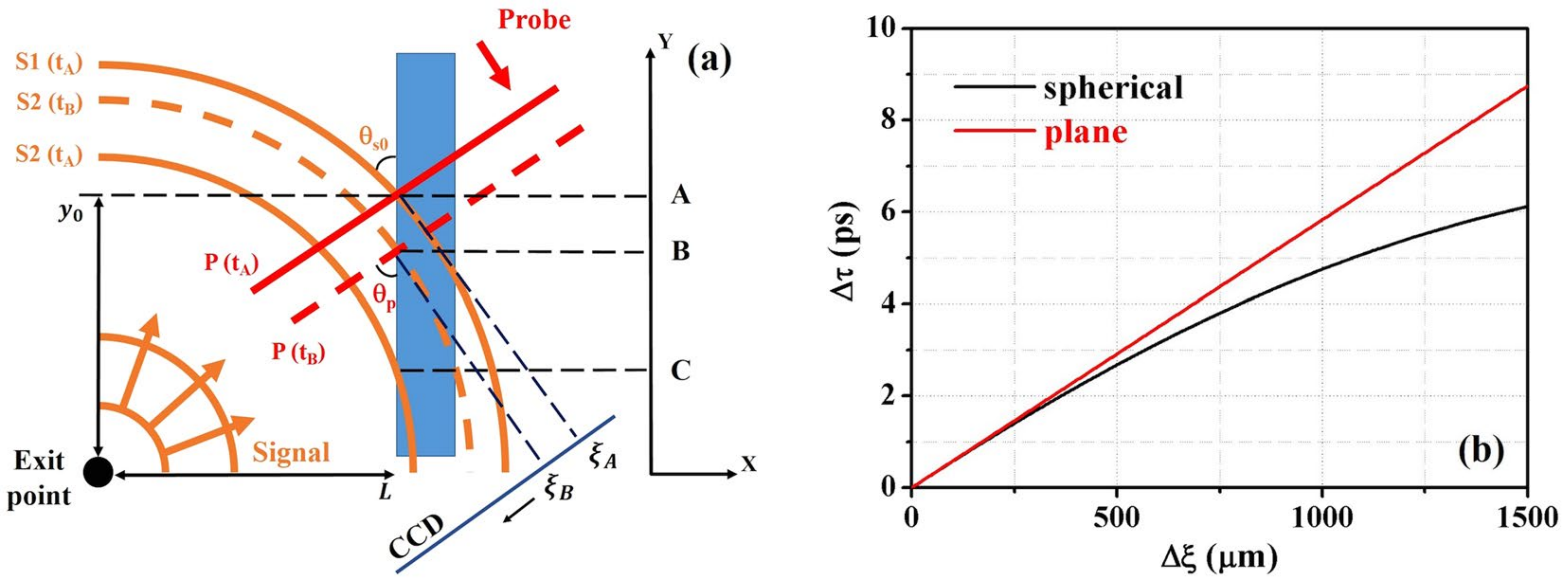
Nonlinear temporal mapping relationship. (**a**) is the calculation frame of the spherical wavefront signal where the blue rectangular area denotes the EO crystal, orange spherical curves represent the Coulomb signal lines and red lines denote the probe wavefront. The probe and first signal line have incident angles of *θ*_*p*_ and *θ*_*s*0_, respectively. At time *t*_*A*_, S1 collides with the probe on the crystal front surface at A, S2 crosses the crystal at C. At *t*_*B*_, S2 collides with the probe on the crystal front surface at B. (**b**) illustrates the calculated temporal mapping relationship for both spherical and plane wavefront signal cases.

Since the spherical shape of the Coulomb field wavefront cannot be neglected at large observation angles, a new temporal mapping relationship should be derived for the application of EO spatial decoding diagnostics in laser plasma acceleration experiments. The calculation geometry of spherical signal wavefronts is illustrated in Fig. 6(a). We consider two signal lines S1 and S2 in the Coulomb signal field with a time delay of Δ*τ*. The probe laser collides with the first signal line S1 at time *t*_*A*_ (point A on Y axis) and second signal line S2 at time *t*_*B*_ (point B on Y axis) on the crystal front surface. The distance between the source position to the crystal front surface is *L*. The cross points of signal lines S1 and S2 with the crystal front surface have displacement functions of: *y*_*s*1_(*t*) = $\sqrt{{c}^{2}{t}^{2}-{L}^{2}}$ and *y*_*s*2_(*t*) = $\sqrt{{c}^{2}{(t-{\rm{\Delta }}\tau )}^{2}-{L}^{2}}$, respectively. The probe laser and crystal front surface have a cross point at *y*_*p*_(*t*) = *y*_*p*0_ − *ct*/sin *θ*_*p*_, where *y*_*p*0_ is the initial probe position that originates from the probe laser delay settings.

At time *t*_*A*_, we set *y*_0_ = *y*_*s*1_(*t*_*A*_) = *y*_*p*_(*t*_*A*_) and define the signal incident angle to be *θ*_*s*0_ = tan^−1^(*y*_0_/*L*) (In the experiment, *y*_0_ was choosen to be at the center of the probe laser spot by setting a suitable probe delay). Thus, we have the initial probe position *y*_*p*0_ = *L* tan *θ*_*s*0_ + *ct*_*A*_/sin *θ*_*p*_, and the timing *t*_*A*_ is determined by *ct*_*A*_ = *L*/cos *θ*_*s*0_.

At time *t*_*B*_ (${t}_{B} > {t}_{A}$), the colliding position of the probe laser with the second signal line S2 can be determined by *y*_*p*_(*t*_*B*_) = *y*_*s*2_(*t*_*B*_), that is: $\,{y}_{p0}-\frac{c{t}_{B}}{\sin \,{\theta }_{p}}=\sqrt{{c}^{2}{({t}_{B}-{\rm{\Delta }}\tau )}^{2}-{L}^{2}}$. We observe the signal displacement Δ*ξ* by projection of the displacement of the two cross points (distance between A and B) to the CCD chip:

3 $${\rm{\Delta }}\xi =({y}_{p}({t}_{A})-{y}_{p}({t}_{B}))\cos \,{\theta }_{p}=c({t}_{B}-{t}_{A})/\tan \,{\theta }_{p}$$

Based on the discussions above, we have the equation determining the temporal mapping relationship as:

4 $$L\,\tan \,{\theta }_{s0}-\frac{{\rm{\Delta }}\xi }{\cos \,{\theta }_{p}}=\sqrt{{({\rm{\Delta }}\xi \tan {\theta }_{p}+\frac{L}{\cos {\theta }_{s0}}-c{\rm{\Delta }}\tau )}^{2}-{L}^{2}}$$

Eq. (4) has solutions: $c{\rm{\Delta }}\tau ={\rm{\Delta }}\xi \,\tan \,{\theta }_{p}+\frac{L}{\cos \,{\theta }_{s0}}\pm \frac{L}{\cos \,{\theta }_{s0}}\sqrt{1-\frac{2\,\sin \,{\theta }_{s0}\,\cos \,{\theta }_{s0}}{\cos \,{\theta }_{p}}\frac{{\rm{\Delta }}\xi }{L}+\frac{{\cos }^{2}{\theta }_{s0}}{{\cos }^{2}{\theta }_{p}}\frac{{\rm{\Delta }}{\xi }^{2}}{{L}^{2}}}$. When we observe zero displacement on the CCD: Δ*ξ* → 0, the timing difference should be zero: *c*Δ*τ* → 0. We choose the “–” solution:

5 $$c{\rm{\Delta }}\tau ={\rm{\Delta }}\xi \,\tan \,{\theta }_{p}+\frac{L}{\cos \,{\theta }_{s0}}-\frac{L}{\cos \,{\theta }_{s0}}\sqrt{1-\frac{2\,\sin \,{\theta }_{s0}\,\cos \,{\theta }_{s0}}{\cos \,{\theta }_{p}}\frac{{\rm{\Delta }}\xi }{L}+\frac{{\cos }^{2}{\theta }_{s0}}{{\cos }^{2}{\theta }_{p}}\frac{{\rm{\Delta }}{\xi }^{2}}{{L}^{2}}}$$

This is the nonlinear temporal mapping relationship in the geometry of spherical-wavefront signal. If we invesitigate on a small time scale such as the case in LWFA, where ${\rm{\Delta }}\xi \ll L$, we can expand the Eq. (5) as $c{\rm{\Delta }}\tau =(1+\frac{\sin \,{\theta }_{s0}}{\sin \,{\theta }_{p}})\tan \,{\theta }_{p}{\rm{\Delta }}\xi -\frac{\cos \,{\theta }_{s0}}{{\cos }^{2}{\theta }_{p}}\frac{{\rm{\Delta }}{\xi }^{2}}{2L}$. Ignoring the higher order term, we get a linear equation showing the case of an obliquely incident plane wave signal:

6 $$c{\rm{\Delta }}\tau =(1+\frac{\sin \,{\theta }_{s0}}{\sin \,{\theta }_{p}})\tan {\theta }_{p}{\rm{\Delta }}\xi $$

By setting *y*_0_ = 1.5 mm, L = 2.2 mm, Eq. (5) and Eq. (6) are plotted out in Fig. 6(b) as the black curve and red line, respectively. We see that, for a measurement with large timing difference, the plane wave assumption has larger timing delay than the realistic value and the data should be fitted with a nonlinear relationship based on the spherical-wavefront field. Yet, for small timing gap in the Coulomb field, the plane wave assumption could be applicable because the difference of the calculated values between both cases is very small. As shown in Fig. 3(e), the measured temporal mapping relationship fitted well with a linear fitting. The average signal incident angle can be calculated from Eq. (6) to be $\bar{{\theta }_{s}}=33.1$° according to the fitted relationship in Fig. 3(e), which is in correspondence with the experimental set up of *θ*_*s*0_ = tan^−1^(1.5 mm/2.2 mm) = 34.3°. This correspondence demonstrates that the Coulomb field has a spherical wavefront structure and the derived temporal mapping relationship denoted by Eq. (6) is applicable in the large observation angle set-up for detecting the temporal information of electron beams from LWFA. Furthermore, we find that, if *θ*_*s*0_ = 0 and ${\rm{\Delta }}\xi \ll L$, Eq. (6) returns to *c*Δ*τ* = Δ*ξ*tan*θ*_*p*_, which corresponds to the case of a conventional accelerator where the EO crystal is placed far away from the source and the observation angle is very small.

## Discussion

**Figure 7:**
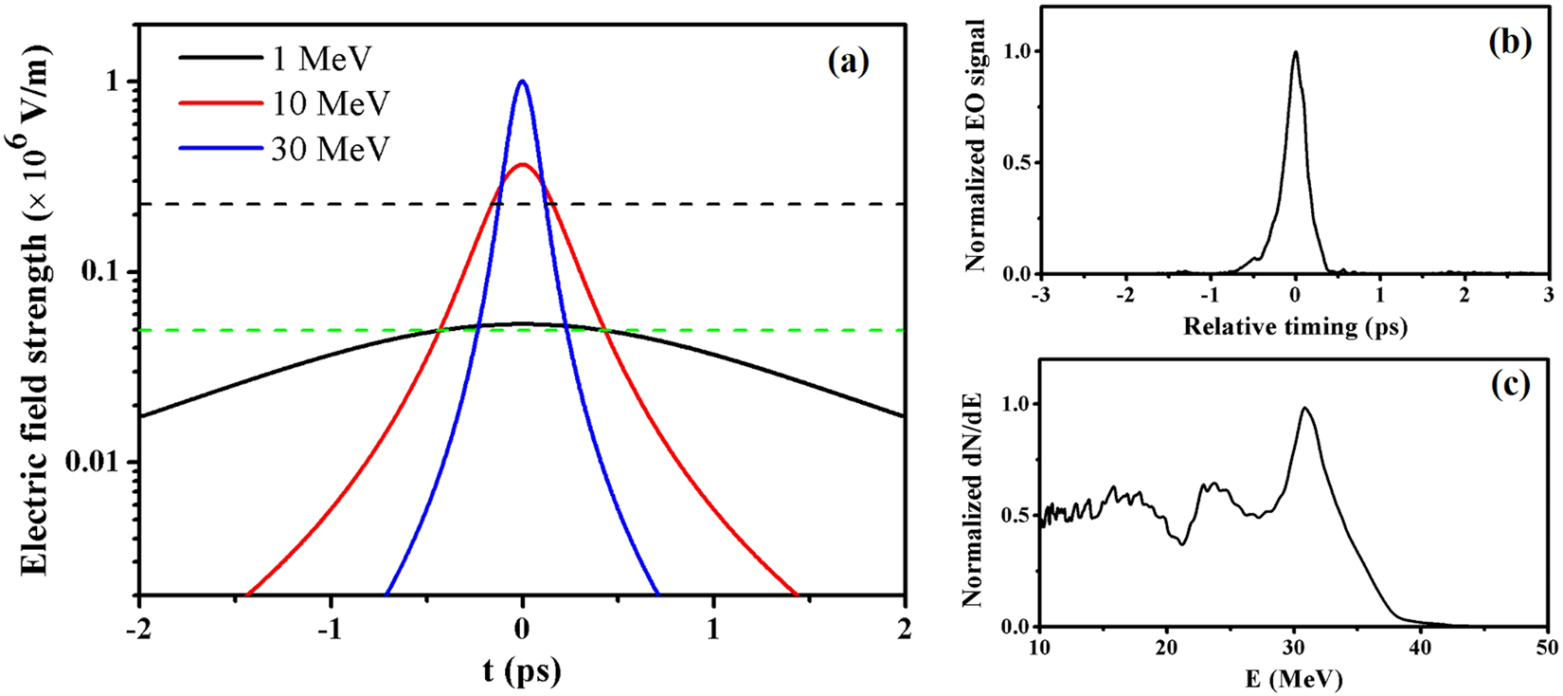
Discussions. The estimated Coulomb field strength and duration are illustrated in (**a**). The blue, red and black curves represent the calculated Coulomb field temporal profile of the electron beams with energy of 30 MeV, 10 MeV and 1 MeV, respectively. The estimated detection threshold was denoted by the black dashed line. The green dashed line represents a field strength of 5 × 10^4^ V/m. (**b**) and (**c**) show a typical EO signal line-out and a simultaneously detected electron energy spectrum (numbers per MeV) at a plasma density of 3.5 × 10^19^ cm^−3^, respectively. The signal intensities in both figures have been normalized. The zero timing in (**b**) is determined as the mean peak EO signal position of 20 consecutive shots. A Gaussian fitting of the line-out in (**b**) shows a duration of *σ* = 115 fs (rms).

The electric field of the electron beam can be roughly estimated as a convolution *E*_*THz*_ = (*E* * *Q*)(*r*, *t*)^33^, where *E*(*t*) = $\frac{e}{4\pi {\varepsilon }_{0}}\frac{\gamma r}{({r}^{2}+{\gamma }^{2}{v}^{2}{t}^{2}{)}^{3/2}}$ is the radial electric field of a single electron at distance *r*, *Q*(*t*) = $\frac{1}{\sqrt{2\pi {\sigma }^{2}}}\exp (-{t}^{2}/2{\sigma }^{2})$ is the electron beam temporal distribution assuming a Gaussian distribution and *v* is the electron velocity. It is related to the electron energy and three dimensional charge density. Assuming an electron beam has infinitesimal transverse size with a pulse duration of 20 fs (rms) and charge of 20 pC (i.e.1 kA), the Coulomb field temporal profiles of various electron energies at transverse distance r = 1.5 mm are calculated and plotted in Fig. 7(a). We see that the electric field becomes weaker and broader with lower electron energy since the the field is compressed with the ratio of ∝ 1/*γ*^37^. The peak electric field strengths of 30 MeV, 10 MeV, and 1 MeV electron beams are 1.0 × 10^6^ V/m, 3.7 × 10^5^ V/m and 5.3 × 10^4^ V/m, respectively. Since the detected signal ${I}_{sig}\propto {\sin }^{2}({\rm{\Gamma }}\mathrm{/2)}\propto {E}_{T\,Hz}^{2}$ in the cross-polarization set-up^33^, where Γ ∝ *E*_*THz*_ is the phase retardation, the signal intensity of the 30 MeV electron beam should be 7 times and 356 times larger than 10 MeV and 1 MeV ones, respectively. In the experiment, when quasi-monoenergetic electron beams with a peak energy around 30 MeV (as shown in Fig. 2(d)) are generated (average charge of 17 pC), the EO signals have an average peak intensity of ∼ 30000 counts on the 16 bit CCD, while the background was at level of 1600. Based on the calculation, the signal from a 10 MeV electron beam is just above the detection threshold and the signal from a 1 MeV beam is undetectable, assuming the same current of 1 kA.

We want to clarify that the CTR emission did not affect our EO measurement. CTR in the THz range could be generated when the electrons pass the plasma vacuum boundary. However, since the underdense gas profile is much more gentle than a solid metal surface, the intensity of CTR should be rather weak. The CTR emission is concentrated at an angle of 1/*γ* aside from the forward direction^39^. For higher energy electron beams, the CTR emission direction is very close to the laser direction so that it cannot be detected in our experimental set-up with an observation angle of 34.3°. For CTR generated by hot electrons, experimental measurement has been made by J. van Tilborg *et al*.^19^, where similar plasma densities were used and nC electron beams with Maxwellian energy distribution were generated. According to the experimental set-up and results therein, the THz field strength could be roughly estimated to be <5 × 10^4^ V/m at the detection point in our experiment. The CTR field strength should be at a similar level or smaller than the Coulomb field of a 1 kA, 1 MeV electron beam and much lower than the detection threshold.

Using the measured temporal mapping relationship illustrated in Fig. 3(e), we found that the durations of the EO signals in Fig. 3 resided between 110 fs–150 fs (rms). A typical signal profile with duration of 115 fs (rms) is plotted out in Fig. 7(b). Figure 7(c) shows the corresponding electron energy spectrum. The observed EO signal durations were much larger than the plasma wakewave timescale (∼ 20 fs). One of the main reasons is that the electron beams generated in our experiment had relatively low energies. As shown in Fig. 7(a), the Coulomb fields of 30 MeV and 10 MeV electrons expand to 70 fs and 188 fs (rms), respectively. The measured EO signal duration illustrated in Fig. 7(b) is in correspondence with the simultaneously detected electron energy spectrum in Fig. 7(c) where a low energy tail existed. Other effects such as: crystal absorption, phase mismatch because of the difference between THz phase velocity and probe laser group velocity and smearing caused by multi bunches will also limit the resolution of the measurement^33^. A better resolution can be achieved by measuring electron beams with higher energies since the Coulomb fields at the detection position could possess temporal distributions similar to those of the electron bunches. The directly observable pulse duration using a GaP crystal has been calculated to be >50 fs (RMS) with 450 MeV electron bunches in previous numerical studies^33,40^. Although direct observation of several femtosecond pulse duration is difficult, such a set-up can still serve well as an electron bunch timing monitor even for electron beams with relatively lower energies since the peak position of the field is not affected^30,31^. On the other hand, the observed broadened signal also demonstrated that the main contributions to the EO signals came from the Coulomb fields of the electron beams. The CTR emissions from electron beams should possess the original electron pulse duration and propagate in the form of electro-magnetic waves. If CTR contributed considerably to the signal, the observed signal should be on a level of several tens of femtoseconds^19^.

In summary, the EO spatial decoding diagnostic was introduced to LWFA. By placing the EO crystal very close to the electron source, we found the measured temporal mapping relationship was different from that used in previous work. Analytic calculations and simulations demonstrated that the Coulomb fields of the electron beams have spherical wavefronts due to the shielding of the plasma. A general nonlinear temporal mapping relationship was derived in a geometry where the signals had spherical wavefronts. This study could be helpful for applications of the EO spatial decoding method in laser plasma acceleration experiments.

## Methods

### Laser system and electron generation

The experiment was performed on the JLITE-X Ti: Sapphire laser system at Kansai Photon Science Institute, National Institutes for Quantum and Radiological Science and Technology (QST), Kyoto, Japan. In the experiment, the 10 TW laser pulses with pulse durations of 40 fs (FWHM) were focused using an F/20 off-axis-parabolic (OAP) mirror and incident on a 3 mm supersonic conical Helium gas jet to generate relativistic electron beams. The focal spot has a 1/*e*^2^ radius of *w*_0_ = 21 *μ*m. The resultant laser peak intensity was I = 1.6 × 10^18^ W/cm^2^, corresponding to a normalized vector potential ${a}_{0}\sim 0.87$, where *a*_0_ = 8.6 × 10^−10^*λ*[*μ*m] I^1/2^ [W/cm^2^]. LWFA in our experiment was performed with a moderate power laser in the case where *a*_0_ < 1. Collimated electron beams were generated when the plasma density was > 2 × 10^19^ cm^−3^. The neutral gas densities have been measured by a Mach-Zehnder interferometer.

### Simulations

We performed two-dimensional simulations using the PIC code REMP in two modes. Firstly, we considered the laser pulse interaction with a supersonic jet modeled as a plasma slab with a bell-shaped longitudinal profile of electron density. The maximum density at the center of the plasma slab was set to be *n*_*e*_ = 1.7 × 10^19^ cm^−3^. The maximum-to-periphery density ratio was 3. The laser pulse with the energy of 0.5 J had a Gaussian spatial profile both in longitudinal and transverse directions with a corresponding FWHM width of 15 *λ* and 26 *λ* respectively, where *λ* = 800 nm is the laser wavelength. Its focal plane was set at the depth about 1000 *λ* inside the density slab. The total length of the plasma slab was 2444 *λ*. The simulation was performed within a window moving at the speed of light along the laser axis with a size of 512 *λ* × 1024 *λ*. The simulation resolution was *λ*/32 × *λ*/16. The total number of quasiparticles was 1.6 × 10 ^9^. The laser pulse propagated through the plasma producing several relativistic electron bunches with energies > 50 MeV. The electron bunches escaping from the plasma were accompanied by their Coulomb field with a characteristic spherical distribution.

For the second mode, we performed a simulation of an isolated electron bunch detaching from a neutralizing background. The electron bunch with a quasiparticle number of 10^5^ was emitted from the left boundary. Electrons were set at rest when born and gradually accelerated to 30 MeV within 100 *μ*m. A moving window with a size of 1.5 mm × 4 mm was utilized. The spatial resolutions were Δ*x* = Δ*y* = 1/16 *μ*m. A Gaussian spatial distribution was assumed with an initial transverse and longitudinal bunch size of *σ*_*x*_ = 50 *μ*m and *σ*_*y*_ = 20 *μ*m, respectively. Divergence in terms of *p*_*y*_/*p*_*x*_ was set to be 1%. The EO crystal was modeled as a rectangular area with an assumed dielectric constant of *ε* = 9. The imaginary part in the refractive index was assumed to be zero. The EO crystal was placed at the same position as in the experiment.

## References

[1] Tajima T, Dawson JM (1979). Laser electron accelerator. Physical Review Letters.

[2] Pukhov A, Meyer-ter-Vehn J (2002). Laser wake field acceleration: the highly non-linear broken-wave regime. Applied Physics B: Lasers and Optics.

[3] Lu W, Huang C, Zhou M, Mori WB, Katsouleas T (2006). Nonlinear theory for relativistic plasma wakefields in the blowout regime. Physical Review Letters.

[4] Faure J (2004). A laser-plasma accelerator producing monoenergetic electron beams. Nature.

[5] Mangles SP (2004). Monoenergetic beams of relativistic electrons from intense laser-plasma interactions. Nature.

[6] Geddes CGR (2004). High-quality electron beams from a laser wakefield accelerator using plasma-channel guiding. Nature.

[7] Leemans WP (2006). GeV electron beams from a centimetre-scale accelerator. Nature Physics.

[8] Wang X (2013). Quasi-monoenergetic laser-plasma acceleration of electrons to 2 GeV. Nature Communications.

[9] Leemans WP (2014). Multi-GeV electron beams from capillary-discharge-guided subpetawatt laser pulses in the self-trapping regime. Physical Review Letters.

[10] Kim HT (2013). Enhancement of electron energy to the multi-GeV regime by a dual-stage laser-wakefield accelerator pumped by petawatt laser pulses. Physical Review Letters.

[11] Wang WT (2016). High-brightness high-energy electron beams from a laser wakefield accelerator via energy chirp control. Physical Review Letters.

[12] Nakajima K (2008). Compact X-ray sources: Towards a table-top free-electron laser. Nature Physics.

[13] Fuchs M (2009). Laser-driven soft-X-ray undulator source. Nature Physics.

[14] Rousse A (2004). Production of a keV X-ray beam from synchrotron radiation in relativistic laser-plasma interaction. Physical Review Letters.

[15] Kando M (2009). Enhancement of photon number reflected by the relativistic flying mirror. Physical Review Letters.

[16] Powers ND (2013). Quasi-monoenergetic and tunable X-rays from a laser-driven Compton light source. Nature Photonics.

[17] Ta Phuoc K (2007). Demonstration of the ultrafast nature of laser produced betatron radiation. Physics of Plasmas.

[18] Albert F (2014). Laser wakefield accelerator based light sources: potential applications and requirements. Plasma Physics and Controlled Fusion.

[19] Van Tilborg J (2006). Temporal characterization of femtosecond laser-plasma-accelerated electron bunches using terahertz radiation. Physical Review Letters.

[20] Lundh O (2011). Few femtosecond, few kiloampere electron bunch produced by a laser-plasma accelerator. Nature Physics.

[21] Debus AD (2010). Electron bunch length measurements from laser-accelerated electrons using single-shot THz time-domain interferometry. Physical Review Letters.

[22] Yan X (2000). Subpicosecond electro-optic measurement of relativistic electron pulses. Physical Review Letters.

[23] Wilke I (2002). Single-shot electron-beam bunch length measurements. Physical Review Letters.

[24] Berden G (2004). Electro-optic technique with improved time resolution for real-time, nondestructive, single-shot measurements of femtosecond electron bunch profiles. Physical Review Letters.

[25] Berden G (2007). Benchmarking of electro-optic monitors for femtosecond electron bunches. Physical Review Letters.

[26] Steffen B (2009). Electro-optic time profile monitors for femtosecond electron bunches at the soft x-ray free-electron laser FLASH. Physical Review Special Topics-Accelerators and Beams.

[27] Shan J (2000). Single-shot measurement of terahertz electromagnetic pulses by use of electro-optic sampling. Optics Letters.

[28] Cavalieri AL (2005). Clocking femtosecond X rays. Physical Review Letters.

[29] Yang X (2009). Electron bunch length monitors using spatially encoded electro-optical technique in an orthogonal configuration. Applied Physics Letters.

[30] Wang W (2017). Temporal profile monitor based on electro-optic spatial decoding for low-energy bunches. Physical Review Accelerators and Beams.

[31] Scoby CM (2010). Electro-optic sampling at 90 degree interaction geometry for time-of-arrival stamping of ultrafast relativistic electron diffraction. Physical Review Special Topics-Accelerators and Beams.

[32] Jamison SP (2006). Electro-optic techniques for temporal profile characterisation of relativistic Coulomb fields and coherent synchrotron radiation. Nuclear Instruments and Methods in Physics Research Section A: Accelerators, Spectrometers, Detectors and Associated Equipment.

[33] Steffen, B. Electro-optic methods for longitudinal bunch diagnostics at FLASH, Thesis, *The University of Hamburg* (2007).

[34] Sütterlin D (2010). Single-shot electron bunch length measurements using a spatial electro-optical autocorrelation interferometer. Review of Scientific Instruments.

[35] Pompili R (2016). Femtosecond dynamics of energetic electrons in high intensity laser-matter interactions. Scientific Reports.

[36] Li Y (2011). Experimental study on GaP surface damage threshold induced by a high repetition rate femtosecond laser. Applied Optics.

[37] Jackson, J. D. *Classical electrodynamics*. John Wiley & Sons (2007).

[38] Esirkepov TZ (2001). Exact charge conservation scheme for particle-in-cell simulation with an arbitrary form-factor. Computer Physics Communications.

[39] Faure J (2006). Ultrashort laser pulses and ultrashort electron bunches generated in relativistic laser-plasma interaction. Physics of Plasmas.

[40] Casalbuoni S (2008). Numerical studies on the electro-optic detection of femtosecond electron bunches. Physical Review Special Topics-Accelerators and Beams.

